# Autism, Stigma, and South Asian Immigrant Families in Canada

**DOI:** 10.3390/ijerph21030369

**Published:** 2024-03-19

**Authors:** Fariha Shafi, Amirtha Karunakaran, Farah Ahmad

**Affiliations:** School of Health Policy and Management, York University, Toronto, ON M3J 1P3, Canada; farihash@yorku.ca (F.S.); ami_k27@hotmail.com (A.K.)

**Keywords:** autism, stigma, South Asian, immigrant families, parent experiences, services access

## Abstract

Considerable empirical evidence suggests early recognition of autism and access to support result in long-term positive outcomes for children and youth on the spectrum and their families. However, children of racialized families are often diagnosed at later ages, are more likely to be misdiagnosed, and experience many barriers to service access. There is also a paucity of research exploring the experiences of parents from specific immigrant groups caring for their children on the spectrum in Canada, many of whom identify as members of racialized communities. As such, the main aim of the study was to examine how South Asian immigrant parents in Canada are experiencing available care programs and support. Another aim was to examine their perceptions of social stigma associated with autism. We conducted an inductive thematic analysis of qualitative data from nine interviews with South Asian parents living in Ontario, Canada. Findings confirmed barriers to an autism diagnosis and to service access. Additionally, parents reported pronounced autism stigma, which enacted impediments to timely diagnosis, service access, and health-promoting behaviors. Findings also revealed that parents experience considerable caregiver stress and psychological distress. The generated evidence is anticipated to inform equitable policy, programming, and practices that better support the needs of children on the spectrum and their immigrant families.

## 1. Introduction

Although autism, a developmental disability, may exist across all genders, ethnic, and socioeconomic groups [1], the experiences of its magnitude vary depending on societal responses to it [2]. Studies conducted in developed countries report that immigrant and racialized families caring for their family members on the autism spectrum experience a multitude of challenges in accessing support and services, along with judgmental attitudes from service providers and their communities [3,4,5,6,7]. This adds a layer of complexity as a wide range of support and services are needed, given the heterogeneity that exists in abilities, traits, and characteristics of people on the autism spectrum, which also vary across life stages and functional domains [8]. However, limited scholarly knowledge exists about the experiences of specific immigrant or racialized groups in Canada. We present here the challenges often reported by families and caregivers of children and youth on the spectrum in the US and Canada, followed by a handful of studies with specific racialized and immigrant groups, calling for further scholarly work primarily in the Canadian context.

Previous studies show that families of children on the spectrum frequently report difficulties in obtaining an accurate early diagnosis [9], long wait times for diagnosis and access to early-age support programs [10,11], a lack of access to trained service providers [12], and obstacles to obtaining information about available support and services [13], among others. In addition, access to an autism spectrum diagnosis in itself appears to be stratified along intersecting socioeconomic, racial, ethnic, and geographic strata [14,15], such that studies examining autism in the US suggest that children of racialized and other visible minority groups who tend to be diagnosed at older ages [15] are more likely to be misdiagnosed [16] and or diagnosed after the age of four compared to their non-immigrant peers [17].

Stigma is another challenge. Goffman defined stigma as an “attribute that is deeply discrediting” [18] (p. 3). Despite discrediting attributes not being negative by their very nature [18], they are constructed or interpreted systematically as such by a society that views disabled individuals as deviant or inferior [19] and, in general, locates individuals within a hierarchy of traits that decides their allocation of privilege, status, and power [20]. As such, certain disabilities in society are more stigmatized than others, with neurodivergent individuals, including those on the spectrum, being one of the most highly stigmatized of all groups [21]. Another widely used conceptualization of stigma distinguishes between felt and enacted stigma. Felt stigma entails beliefs that individuals might encounter negative treatment from others if their stigmatized condition is known, along with internalization of shame and/or fear of discrimination that may deter help-seeking. In contrast, enacted stigma refers to experiences of unfair treatment or discrimination toward stigmatized individuals [22,23,24].

At the same time, stigma is not limited to children on the spectrum alone. Families caring for children on the spectrum face judgmental attitudes embedded in systems they interact with every day [24,25]. In many instances, family members are subjected to prejudice and discrimination because of their association with individuals living with disabilities [18,26], also referred to as courtesy stigma [24]. Family members may also be shamed by others as if they have caused a child’s disability [25] or are responsible for the behaviors of children, often with invisible disabilities, as is sometimes the case with autism spectrum due to perceived inferior parenting [24]. Such attributions and treatment can lead family members to develop negative self-evaluations and negative emotions [27]. For instance, in a 2016 study that recruited parents of children on the spectrum from the United States (US) and Canada, 40.4% of the parents reported distancing themselves from friends and family, frequently or sometimes within the past 6 months, due to their child’s autism-related behavior [28].

The challenges are likely more severe for immigrant or racialized families, even in economically developed countries such as Canada, given the high levels of stigma around disability in certain countries [29,30] and that immigrants often have to learn to navigate complex, new systems in their adopted countries amidst language and communication barriers [5,6], and may also experience social isolation and a lack of support due to their immigrant status and physical distance from family [6,31]. However, there is a dearth of research on the experiences of parents from specific immigrant or racialized groups such as South Asians. This is concerning, given Canada’s growing cultural and racial diversity due to ongoing immigration. In 2021, more than 8.3 million people, or almost one in four people (23.0%), were or had been landed immigrants or permanent residents in Canada [32]. Further, according to the 2021 census, South Asians represent the largest visible minority group in Canada [33].

In fact, in the Canadian context, only a handful of Canadian studies report on the experiences of South Asian immigrant parents. In 2017, Khanlou et al. recruited 21 immigrant mothers of children on the spectrum from diverse ethnocultural backgrounds, including those of South Asian backgrounds with English fluency, for a qualitative study in Toronto, Canada. They reported families’ service-access barriers like delays in obtaining a concrete diagnosis, language and communication challenges, lack of information about care programs, and economic hardships around having to secure out-of-pocket expensive services given the underfunding of government programs and a lack of comprehensive coverage for services [5]. Grewal in their study, explored the experiences of eight South Asian parents with fluency in English navigating an autism spectrum diagnosis and support and services in British Columbia and Alberta. Alongside barriers around communication challenges and government funding constraints, parents in their study also reported cultural barriers such as feeling judged by others within the community and a lack of community knowledge of autism [34]. In a more recent study, Shanmugarajah et al. recruited Tamil mothers caring for children on the spectrum in Toronto and reported similar access barriers [6]. However, all these studies either limited their inclusion criteria to only parents with comprehension of the English language and/or conducted the interviews in English, therefore fully precluding the experiences of some immigrant parents while not capturing the breadth and depth of experiences and perspectives of others. Notably, none examined the experiences or perception of stigma associated with the developmental disability of autism.

The social model of disability posits disability as a socially constructed phenomenon and highlights the distinction between impairments that are socially valued differences versus disabilities that are created because of the inability or unwillingness of society to accommodate such impairments [35]. Stigma is thus perceived as a societal unwillingness to accommodate people with traits other than “normal”. Further, understanding disability as a social construct necessitates that analysis be expanded to also include people without a disability, as much of the meaning assigned to disability originates from them [36]. Moreover, the support available within an environment can either make it an enabling or a disabling experience for those living with disabilities [37]. As such, there is a need to advance scholarly knowledge by examining the experiences of parents of specific immigrant and racialized groups through a social disability lens and stigma to better inform practices and policy making.

With this aim, we planned a study with South Asian immigrant families navigating autism-related services in Canada and offered participation in English, Urdu, Hindi, or Tamil languages. The two-fold aims of the study were to elicit experiences and perceptions of South Asian immigrant parents in Canada about autism care programs and support for their child on the spectrum and to examine social stigma.

## 2. Methods

The study was conducted using in-depth one-to-one qualitative interviews [38,39]. This approach is considered suitable for sensitive topics as it offers privacy to the participants and enhances the interviewer’s ability to gauge and address any discomfort a participant may experience when sharing their experiences and views [39]. Ethical approval for the study was obtained from the research ethics committee at York University.

The study design was informed by the critical social science perspective whereby reality is built socio-historically, and the status quo is transformable as humans have agency and dynamically interact with their social and living conditions [40]. Scholarly work informed by this research paradigm also aims to bring forth social transformation through amplifying subjugated voices and experiences. This was further deepened using the social disability model, which identifies stigma as a societal barrier in need of change. To inform our analysis and interpretation, we then drew from both Goffman’s theoretical work on stigma and Scambler’s concepts of felt and enacted stigma [18,24], as discussed earlier.

### 2.1. Participant Recruitment

The research team first established collaboration with community-based agencies serving families caring for children and youth on the autism spectrum in the Greater Toronto Area. The study collaborators were the South Asian Autism Awareness Centre (SAAAC) and Health Access Thorncliffe Park. They provided feedback on the approach to recruitment and supported reach to potential participants by disseminating the study flyer through their listserv. The participant eligibility criteria included being a parent of a child on the spectrum, at least 18 years of age, self-identifying as a Canadian South Asian (e.g., origins in Bangladesh, India, Nepal, Pakistan, or Sri Lanka), having English, Tamil, Urdu, or Hindi language speaking abilities, and being an Ontario resident seeking services from one of two collaborating community agencies. The distribution of flyers through the listserv first led to low interest from potential participants. Thus, we also employed snowballing and encouraged participants to share information in their network, which led to one participant sharing the flyer through a WhatsApp group, facilitating recruitment. Nine eligible parents participated in the interviews.

### 2.2. Interview Procedures

The qualitative interviews were guided by an interview guide with open-ended questions and probes, aiming to generate rich information. For instance, when parents were asked about their initial thoughts and feelings around their child’s diagnosis, they were probed on their acceptance, rejection, or confusion around the diagnosis, knowledge about autism, and community attitudes towards disability, among others. The guide was revised after the first interview by adding probes. This allowed the interviewers (AK and FA) to engage participants in a conversational dialogue around the main topics while probing as needed. The interviewers’ fluency in the first languages spoken by the participants and their interdisciplinary training and experience in the fields of public health, nursing, medicine, and social sciences facilitated trusting relationships and rapport with the participants. AK has taken graduate-level courses in research methods and has conducted research with racialized mothers of children with developmental disabilities. FA conducts research with immigrant and racialized communities using qualitative approaches. Before conducting the interviews, AK engaged in practicing the interview strategies with a community collaborator and FA. The topics of the interview centered on the parents’ experiences and feelings about the process of diagnosis, community attitudes about the child’s disability, and availability of support. Before conducting the interviews, each participating parent received detailed information about the study over email and/or phone and provided informed consent. Each participant also informed the research team about their language preference for the interview. The interviews were organized at a time and day convenient to the participants and conducted online, given pandemic restrictions. Each interview lasted for approximately an hour. Participants also completed a few demographic questions and consented to an audio recording of the interview and taking field notes. A member check was embedded within each interview, where the interviewing researcher summarized the discussion after each topic and asked the participant to add or correct any details. The audio-recorded data were subsequently transcribed verbatim in English by bilingual interviewers and anonymized for analysis.

### 2.3. Qualitative Analysis and Trustworthiness

Data saturation seemed to have occurred after the ninth interview due to attention to context and probing. Qualitative data for the interviews were coded and analyzed via inductive thematic analysis, utilizing a constant comparison technique [41,42,43]. The author (FS) read and re-read the transcripts and started with open coding, which is a process of identifying important words or groups of words in the data and then labeling them based on their meaning. Through teamwork (FS and FA) and constant comparison, the preliminary codes were combined, collapsed, and/or expanded to identify overarching themes and sub-themes. The coding and analysis process was aided by Dedoose software (version 9.0.17). To enhance the trustworthiness of the findings, we closely engaged with the interview data through several rounds of readings, careful line-by-line systematic coding, and team discussions. Informed by the critical social science research paradigm, we also reflected on our social positioning in relation to the research topic and the community being studied so that we could be engaged in critical questions to discount undue influences on the interpretation of findings. One of the authors (FA) has close observation of families caring for their members on the spectrum. She is also a South Asian community member and arrived in Canada as an adult immigrant. AK and FS are also of South Asian background. As such, we all maintained reflexive journals and asked critical questions in our team meetings (for example: Would I interpret it differently if I was not an immigrant or a person with close observations about autism?). Lastly, to enhance transferability, we offered participants narratives with detailed descriptions of the findings.

## 3. Findings

Nine Canadian South Asian parents (two fathers and seven mothers) participated in the qualitative interviews. All were immigrants, and their ages ranged from 29 to 55 years, with a mean of 43 years. Over half of the parents self-identified as Pakistani, while others identified as either Sri Lankan or Bangladeshi. On average, the parents had been in Canada for 14.8 years, with 3 years spent in Canada being the lowest and 26 years being the highest. All parents except one had three children, including the child on the spectrum. Two parents had more than one child on the spectrum. The ages of these parents’ children on the spectrum ranged from 4 to 24 years. The demographic information for the participants is provided in Table 1.

Three major themes identified through inductive qualitative analysis were (1) barriers to service access, (2) stigma, and (3) caregiver stress. These themes and supporting quotations from parent interviews are presented below, with personal identifiers removed to maintain anonymity.

**Theme 1: Barriers to Services Access**

Participant parents reported several barriers in the early phases of trying to obtain an autism spectrum diagnosis and accessing support and services. These included individual, service provider, community, and structural barriers, respectively.

***Individual level:*** Parents reported a lack of knowledge and understanding about autism and language barriers. Their limited knowledge and understanding of autism were markedly prominent, with most interviewees contributing to this sub-theme. Almost all but one of the parents reported that for their children, daycare or preschool teachers or healthcare providers were the first to notice signs of a possible autism spectrum. Only one mother with a psychology background was able to identify on her own. Further, the majority of the parents had never heard of autism or had no family members or relatives with an autism spectrum diagnosis. As such, for many of these parents, receiving an autism spectrum diagnosis was particularly confusing.

*His school suggested that there is a chance for him to have special challenges…. [on the day of diagnosis]. No, it was not [clear]. I had to study a lot.*

*(F1, 55)*

*When he got diagnosed, we did not have a single clue about it because not in my family, not in my husband’s family, do we have anyone with an autism spectrum diagnosis.*

*(M4, 41)*

Given limited autism knowledge, cultural notions of gender-typical behavior appeared to have influenced some parents’ appraisals of the development of their children. Two mothers, for instance, attributed their daughters’ developmental delays to girl children being “shy.”

*With my daughter, she would not respond when we called her by her name. She would only lower her head…. However, we assumed because she is a girl, she is shy.*

*(M2, 29)*

The parents also discussed language barriers in several interviews. Foremost, language barriers made understanding the diagnosis and medical terms particularly challenging for some parents. Moreover, language barriers also prevented parents from accessing certain services available to them or their children. For instance, one parent, not knowing the meaning of “respite” care, was not able to access any such services. When asked if she was accessing respite services, she responded: *I am so sorry, what is respite? (M4, 41)* The language barriers exacerbated their challenges to learn about the child’s developmental condition and services in a timely manner.

*I had a language barrier when they gave me the diagnosis; it was even difficult to understand in Tamil. It took me a long time to understand.*

*(M7, 39)*

*Yes, only the word autism I was able to understand [in initial encounter] and that is it.*

*(M6, 36)*

***Service level:*** While most parents were satisfied with the services they received and spoke positively about their experiences with family physicians and specialists, the use of medical terminologies, lack of interpreters, and limited provider sensitivity towards client needs were discussed in some of the interviews. In one interview, a father narrated the challenges his family experienced because the specialist physician referred to by his family physician would not communicate properly. Similarly, another parent discussed how her child’s specialist physician went on leave after the first day of what was supposed to be a three-day diagnostic assessment; she had to wait for another six to eight months before the assessment could be restarted.

*He [the family physician] gave me another referral and told me to go for that. But unfortunately, that doctor was no longer there. And I did not get any communication from them. Whenever I called them, they did not respond properly.*

*(F1, 55)*

*But after one day’s assessment, she assessed him and after that, she was going to leave—I do not know why—she went on leave, sick leave, or some domestic problem, so she went.*

*(M7, 39)*

*My home language is Urdu, and he [son] understands Urdu and every single direction [I give] he, you know, follows. In English, he has a little difficulty because my husband and I speak Urdu at home all the time. However, there was no interpreter on the day of the diagnosis.*

*(M6, 36)*

The experiences of limited provider sensitivity contributed to distress for some parents. For example, one parent shared the persistent discordance between the family physician and her child’s specialist about whether the child meets the criteria for an autism spectrum diagnosis. Further adding to her confusion and distress, she quotes the family physician as having said: *“No, who is she? I do not agree with her [the specialist physician] because I do not see any symptoms of autism.” (M6, 36)* When approached for a disability tax credit form for her child, the same family physician again did not cooperate, citing that she does not believe the child is on the spectrum.

***Community level:*** Some parents highlighted that in many communities, the knowledge and understanding of autism are limited, which has implications for them in accessing and maximizing support and services for themselves and their children. For instance, one parent mentioned: *“There is no word in Urdu for autism.” (M3, 41)* So, the way she had to explain her child’s diagnosis to her family was by telling them that he had epilepsy and some sort of developmental delay. Another parent gave the example of her son’s classmate, also on the spectrum, and described that his mother, who is of South Asian background as well, has decided not to pursue any support or services despite receiving funding for them as she believes “*the child will improve on his own*”. Thus, the interviewed parent deemed this behavior indicative of a lack of awareness around autism and one that necessitates autism knowledge promotion at the community level. Another parent described how the extended family’s limited autism knowledge prevented her from seeking services for her child earlier.

*With my friends and family, when we did mention the diagnosis, they would say, “Oh, your husband is the shy type; maybe that is the reason?” Due to such comments, we did not act right away.*

*(M7, 39)*

***Structural level:*** Some parents shared concerns about wait times and the lack of information offered by providers when accessing services to obtain a definitive diagnosis for their child and support programs. For instance, one parent who waited eight months for a referral to a neurologist said: *Yes, it was a long wait. And it was too late, and everything was late. (M7, 39)* Another family tried to contact their doctor when their child was three and a half years old but then commented: *We had to wait for 1 ½ years to get several types of appointments. Then, by the time we got the finding, he was 5 years old. (F1, 55)* The wait time issues continued into the post-diagnosis phase, and some commented that they had to wait a long time to access support and services. For instance, one parent discussed having to wait a long time for their adult daughter’s passport program funding, a reimbursement program that allows adults over 18 to participate in their community and live as independently as possible and includes funding for activities such as day programs.

A few participants discussed the difficulties of accessing information from service providers. For example, a mother expressed concerns about how and where to obtain information about autism support and services. When asked what sort of information she would like to be available for parents of children on the spectrum, she replied: *All kinds of information, like when there is some kind of camp or activity as well as information on support and services. Every kind of support. Just age appropriate. (M6, 36)* Moreover, she also recommends that this information come from healthcare providers and places frequented by immigrant families, such as schools.

In the context of financial constraints and a narrow range of services that qualify for government funding, some parents discussed paying out of pocket for services they deem beneficial for their child. However, they could not continue after some time as they could no longer afford them. One father, for instance, took his child to an acrobatic gymnasium, which offered exclusive classes for disabled children twice a week to help with physical movement. However, he was not able to have his child continue the program despite its perceived benefits and commented: *So, I found it quite good. But it was costly. So, yes. I could not continue for more than one year. (F1, 55)* He further commented that waiting for a long time to obtain government funding was frustrating, and some parents were left with no choice but to stop using the support program.

**Theme 2: Stigma**

Through descriptions of experiences, activities, and feelings around their child’s autism spectrum diagnosis, parents reported findings consistent with both felt stigma and enacted stigma.

***Felt stigma*** was prominent when parents shared their child’s diagnosis with others. Parents also exercised caution when going out in public places and meeting new people, while some socially isolated themselves from friends and family. The felt stigma was identified across interviews in modes of censorship, disclosure with cautions, and isolation. In terms of censorship, many parents described routinely engaging in some form of censorship around their child’s autism spectrum diagnosis. Several mentioned avoiding having to share their child’s diagnosis, even with family members. The reasons for deciding not to share the diagnosis with immediate or extended family included fear of negative attitudes of the community towards disability and unwarranted sympathies.

*If someone does something weird or different similar to what those on the spectrum do, they [family members back home] always say, “Oh he’s mad, he doesn’t know anything.”*

*(F2, 52)*

*The reason for not disclosing is that people start to sympathize, and it does not feel good. They ask a lot of questions; they are not aware of autism.*

*(M3, 41)*

In terms of disclosure with caution, some parents shared the diagnosis only with close friends and family or shared selective information out of necessity. Some examples for the latter included instances when the family was out in public, and their child was experiencing challenges, or when some sort of unsolicited criticism or comment was directed towards their child.

*I would tell them that the kids have autism; they go to a special school and get treatment. I would not go into detail about how this is a life-long problem.*

*(M1, 55)*

*When we go to a concert, program or gathering and someone makes some strange comment, we say he is like that, so accommodate it. It is a way you do not say [he is on the spectrum]. But once something happens, we just try to defend it.*

*(F1, 55)*

Some parents reduced their social networks, and isolation seemed to be their way of reducing felt stigma. These parents shared fears of being judged or past experiences of negative encounters. One mother resorted to prolonged social isolation as she had been terrified of how her relatives would react to her child’s autism spectrum diagnosis.

*Well, we are not very social. Maybe because of my children, I do not feel comfortable. So, the families we meet normally are one or two, and they have known us even before my son’s autism spectrum diagnosis, so they know everything.*

*(M4, 41)*

*Because of this, I kept to myself for years and did not want them [family members] to interact with my child.*

*(M7, 39)*

***Enacted stigma*** also emerged in parents’ shared experiences. While parents were not probed about their experiences of stigma enacted by others, nevertheless, some did share accounts of overt discrimination. For example, a mother underlined the reason why she cannot always take her child out in public, and a father described how a school principal threatened to call the police on his child as the child was experiencing challenges on a particular day:

*We took him to the park or some other area… He grabbed a kid and the kid’s mom said, “You should [be] responsible for your kids. Why did you put him out? You should not take him out because these are our kids, and he grabs and snatches our kids.” They felt so bad. That is why I avoid taking him out in public.*

*(M5, 42)*

*Two years ago, I got a call… His school principal said, “I called the police and I’m calling you, whoever comes faster will take the boy.”*

*(F1, 55)*

It is possible that more parents have had similar experiences but chose not to reference them during the interviews as they were not explicitly asked about overt experiences to mitigate the possibility of traumatization. Equally important, though, is that these findings implicate that factors such as stigma and a lack of social support may mediate the ability of families to optimize support available for their children and themselves alongside exaggerating well-being risks.

**Theme 3: Caregiver Stress**

Without being probed about caregiving stress, parents reported experiencing stress or feelings of anxiety or depression.

*For two years, I did not go out. This contributed to my depression because I kept thinking about what others would think and how they would accept my child’s diagnosis.*

*(M7, 39)*

*The family doctor prescribed me anxiety and depression tablets.*

*(M5, 42)*

When asked about caregiving support received from family, less than half of all parents cited receiving support, and the support they received varied from grandparents looking after the children to friends helping. Among those who did not receive any support at all, it was often because they were unwilling to ask for it or because they had not disclosed the diagnosis to anyone. Stigma also most likely impacted their willingness to disclose. For example, one father, when asked about the support received from the family, mentioned: *We did not ask for any support. (F1, 55)* It is possible that the parent was not even thinking about such a possibility, given the profound disability stigma in the community, as a social disability model would suggest.

While caregiver stress showed up in many ways during the interviews, it was particularly pronounced for one parent who reported having no significant support or caregiving involvement from her partner.

*At this point, I was very fed up with life, and I was lacking the support of my husband, who was not here at the last minute. I was out. I wanted to sort out my son’s care so we could focus and sort out our (husband and wife) problems.*

*(M1, 55)*

Thus, in the absence of caregiving or social support, resources that can be instrumental in coping with demands and stresses associated with parenting a child on the spectrum, it appears that several of these parents had been straining their personal resources to cope and to the point that their well-being was being adversely impacted.

The themes presented, however, are not mutually exclusive. They interact with one another in nuanced, complex ways, shaping the spectrum of experiences and lived realities of these marginalized immigrant parents and their children. For instance, for one parent, numerous challenges in the form of poor knowledge of autism, healthcare professional’s lack of sensitivity towards the family’s needs, and structural barriers such as long wait times translated into delays in obtaining a definitive autism diagnosis, all the while increasing the parent’s feelings of guilt and negatively affecting personally important and meaningful outcomes for her child. For another parent, economic hardship and stigma underlie his family’s experiences trying to navigate health, education, and social services systems in Canada, with stigma in particular, impeding the family’s ability to mobilize social support to facilitate health and well-being, and thereupon entailing implications for not only the child’s development and health but also the parents’ coping abilities, quality of life, and well-being. These findings speak to a multitude of disabling environmental conditions around these families, per social disability model, that need attention through better programming and policies, as discussed below.

## 4. Discussion

The study’s findings contribute to an enhanced understanding of the experiences of South Asian immigrant parents of children on the spectrum, which has remained understudied in the Canadian context. Findings confirm the existence of barriers to an autism spectrum diagnosis and successive access to services and support at the individual, service provider, community, and structural levels. Additionally, findings demonstrate that the stigma of autism in South Asian communities is particularly pronounced and enacts impediments to timely diagnosis, access to services, and health-seeking and health-promoting behaviors for both children on the spectrum and their parents alike. Moreover, findings also reveal that immigrant parents experience considerable caregiver stress and psychological distress. As such, the findings are discussed in more detail below in the context of the existing literature and implications for equity-enhancing future research, programming, and policy.

In our study, participant parents highlighted several barriers to accessing an autism spectrum diagnosis and available support and services. At the individual level, parents discussed limited knowledge and understanding of autism contributing to delays in autism recognition and timely help-seeking for their children, consistent with existing Canadian studies with immigrant families [3,6]. However, the extent of knowledge about autism seems particularly constrained among South Asian parents in our study. A possible reason is the absence of words in their native language to describe autism or developmental delays, leading to a community-level lack of knowledge. Importantly, participant parents were impacted by their community’s low understanding of autism as they became hesitant and anxious to seek support and assistance from extended family and friends. This is of particular concern given that extended family support has been identified as a predictor of a family’s quality of life [44], and scholars also report in other studies that immigrant parents perceive extended family members as having a positive role in the health and well-being of their child on the spectrum [6]. Thus, we argue that not having an adequate understanding of autism, alongside being a community-level barrier, is also a structural barrier and needs to be addressed through system-level approaches to engage and inform the community. A multi-sectoral approach here could be helpful where primary care providers, childcare programs, schools, and religious gathering places are engaged to increase community awareness, especially newcomers, about a child’s developmental milestones and possible delays. In our study, participants’ language and communication challenges often manifested as difficulties in understanding medical terminologies and the diagnosis itself. At the intersections of limited knowledge of the care system and language proficiency, some participants revealed not knowing and accessing “respite services”, which has been previously reported as well [5] and thus speaks volumes about the access inequity among racialized immigrant parents in Canada. In addition, a participant parent in our study described how a lack of an interpreter impacted her son’s diagnostic experience. Collectively, these findings reinstate the need to improve language access for parents and children by providing culturally sensitive and age-appropriate interpretation services.

At the service provider level, some parents expressed dissatisfaction with healthcare providers’ sensitivity to the needs of the families. Other studies with Canadian immigrant families caring for children with disabilities have pointed out the need for healthcare providers to be more sympathetic [5] and patient [4]. Our study adds by also documenting a parent’s distress due to discordance between the family physician and specialist about their child’s diagnosis of autism. The difference in the appraisal of autism spectrum can be explained through research that demonstrates that physicians, including family physicians in Ontario, Canada, tend to cite their undergraduate and postgraduate medical education as inadequate in providing them with sufficient knowledge related to autism [45]; nevertheless, given that the shortage of specialists has been associated with delays in diagnosis [46] and that family physicians often happen to be the first line of contact for children [47], the findings underscore the need to enhance family physicians’ autism-specific training alongside further training on empathy and provision of culturally sensitive care.

Participant parents also expressed concerns about structural level barriers related to wait time for services and financial challenges. Given that parents were many years from the stage of diagnosis, many seemed overall satisfied with the wait time for diagnosis, though some nevertheless expressed frustration. In line with the findings, Rivard et al. have previously reported an average length of 26 months from first concerns to obtaining an autism diagnosis in Quebec, Canada [11]. Shanmugarajah et al. have also documented long wait lists among Tamil mothers in Toronto, Canada, to avail of government funding for autism programs and support [6]. Parents’ financial challenges and socioeconomic inequities influencing the timeliness of an autism diagnosis and access to services have been highlighted by others [48]. There is a strong need to improve policies and practices through a lifespan approach that responds to the needs of children across the spectrum and their primary caregivers, especially those in vulnerable socioeconomic positions.

Stigma was another dominant theme across parents’ interviews. Their discussions on avoiding sharing the diagnosis of their child within their social settings reveal courtesy stigma. Some instances of enacted stigma were also shared. Although studies with immigrant mothers in Canada report some of these insights [5,6], our sample included both fathers and mothers specifically from the South Asian immigrant community, which extends our understanding further. In addition, we found that stigma and fears of it also impact parents’ well-being. For example, parents’ conversations analyzed for this study showed that parents felt social isolation due to their child’s diagnosis as one parent described how the stigma of autism made her keep to herself for years, contributing to her depression. Given the impacts of social stigma on parents and children, and in alignment with the Canadian Academy of Health Sciences identification of social inclusion as a key area that needs to be addressed in the development of a National Autism Strategy [49], much more work is needed for acceptance of difference and disability. A foundational approach would be to employ a disability lens at the societal level from early years (e.g., schools’ curriculum and training of professionals) to workplaces (e.g., inclusive hiring and retention policies) and social domains (e.g., games, sports, and social clubs). The findings also reiterate the need to support families caring for children on the autism spectrum, including South Asian immigrants, through anti-stigma community-based programs and stigma support for parents and caregivers [50,51].

Caregiving stress was also evident in parents’ descriptions of their experiences, feelings, and activities, alongside reports of depression and anxiety. This finding is consistent with previous research that suggests many parents and caregivers of children on the spectrum tend to show clinically significant mental health concerns [52] and frequently report elevated levels of depression and anxiety [53,54]. Although social support has previously been linked with alleviating negative psychological conditions [55] and enhancing well-being [56], in the conversations examined for this study, less than half of the parents received any caregiving support. Among parents who received no support, it was mostly because they had not disclosed their child’s diagnosis to anyone due to fear of stigma. As for immigrants, the primary source of social support is often their own informal social network; hence, programs designed to help strengthen these networks can be instrumental in promoting well-being [57]. In addition, immigrant families can also benefit from the identification and removal of barriers to respite services and increased availability of and access to community-based support groups and mental health services. Finally, the immigrant label encompasses diverse cultural communities from multiple geographic areas. Thus, future research should be planned with larger samples to capture the community-specific needs of newcomers to Canada and develop policies and programs through a systematic equity-based approach for meaningful support.

Lastly, critical analysis of the findings through a social model of disability lens helps illuminate the social nature of disability and the social conditions of disablement. Structural arrangements have a differential effect on immigrant parents versus their non-immigrant counterparts. For instance, government underfunding of autism programs and services and a lack of comprehensive service coverage result in heightened challenges for immigrants with communication challenges that manifest in the form of long wait times for services, delayed diagnosis, and the inability to access respite services. Further, the social disabling conditions interact dynamically, such as service access and parents’ level of income, among others, to create patterns of inequity specific to the context of these parents that aggravate lived experiences of disabilities and caregiving. Moreover, the study findings interpreted through the social disability lens shed light on how the community’s attitudinal barriers contribute to parents’ inability to disclose their children’s diagnosis to even family members or to go out in public with their child on the spectrum without experiencing stress or stigma.

The transferability of the reported findings warrants caution. First, the subjective and contextual nature of findings emerging from qualitative research entails inherent limitations to transferability [39]. Thick descriptions of the participants and research process are provided to mitigate this. Second, the South Asian immigrant participants were recruited through collaborating community organizations in the Greater Toronto Area. Their perspectives and experiences may not represent the views and experiences of those without access to culturally responsive care. Third, on average, the parents had been in Canada for 14.8 years, and only one parent at the time of the interview had been living in Canada for less than 5 years. As such, it was not possible to adequately capture the experiences of new immigrant parents against those who have been in Canada for some time and potentially made necessary adaptations [6]. Future research with a larger sample of new and established immigrant South Asian parents is recommended.

## 5. Conclusions

Our findings reveal that South Asian parents in Canada experience many barriers to an autism spectrum diagnosis and subsequent access to services and support at the individual, service provider, community, and structural levels. We also found a marked stigma of autism in the South Asian community, which impacts help-seeking and health-promoting behaviors and appears to be a risk factor for the poor mental health of racialized immigrant parents caring for their children on the spectrum. Additionally, the parents in our study also reported considerable caregiver stress as a result of limited caregiving help and because of fear of stigma. As such, our findings highlight that equity cannot be attained only through the provision of health, educational, and or social services. Factors such as stigma, social support, social inclusion, structural barriers, and social determinants of health all play a pivotal role in facilitating or inhibiting the ability of parents of children on the spectrum to maximize the resources and services available for their children and themselves.

## Figures and Tables

**Table 1:** Demographic characteristics of the parent participants.

Parent	Age	Education	Cultural Background	Marital Status	Work Status
Father 1	55	Completed university/college	Bangladeshi	Married	Self-employed
Mother 1	55	Up to middle school	Sri Lankan	Divorced	Homemaker
Mother 2	29	Some university/college	Sri Lankan	Married	Self-employed
Father 2	52	Completed university/college	Pakistani	Married	On social assistance
Mother 3	41	Completed university/college	Pakistani	Married	Homemaker
Mother 4	41	Completed university/college	Pakistani	Married	Homemaker
Mother 5	42	Completed university/college	Pakistani	Married	Homemaker
Mother 6	36	Completed university/college	Pakistani	Married	Homemaker
Mother 7	39	Up to high school	Sri Lankan	Married	Homemaker

## Data Availability

The interview data could be made available upon reasonable request to the corresponding author.
